# Horseshoe kidney with triple renal arteries and an aberrant right testicular artery in a male cadaver: case report

**DOI:** 10.1590/1677-5449.202401172

**Published:** 2025-04-18

**Authors:** Dibakar Borthakur, Ritu Sehgal, Neerja Rani, Rajesh Kumar, Rakesh Kumar, Rima Dada

**Affiliations:** 1 All India Institute of Medical Sciences – AIIMS, New Delhi, India.; 2 All India Institute of Medical Sciences – AIIMS, Patna, India.

**Keywords:** horseshoe kidney, accessory renal artery, aberrant testicular artery, rim em ferradura, artéria renal acessória, artéria testicular aberrante

## Abstract

Horseshoe kidney (HSK) is a rare congenital malformation of the kidney with reported prevalence between 1 in 600 and 1 in 400. A typical HSK has two lower poles fused across the midline in front of the abdominal aorta just below the origin of the inferior mesenteric artery. Very rarely, the upper poles may fuse resulting in a reverse HSK. It is more common in males than in females, but no definite racial or genetic predilection has been established. The HSK is frequently associated with other congenital anomalies, the most common being vascular anomalies. This report describes HSK in a 73-year-old male cadaver, with triple renal arteries and aberrant right testicular artery originating from the ipsilateral renal artery and bilateral extra-renal calyces. Although rare in occurrence, a surgeon must bear in mind that such a combination of anomalies may pose technical difficulties during open or laparoscopic abdominal surgeries.

## Resumo

O rim em ferradura é uma malformação congênita rara, com uma prevalência relatada entre 1 em 600 a 1 em 400 casos. Um rim em ferradura típico apresenta dois polos inferiores fundidos ao longo da linha média, à frente da aorta abdominal, logo abaixo da origem da artéria mesentérica inferior. Muito raramente, os polos superiores podem se fundir, resultando em um rim em ferradura reverso. Essa malformação é mais comum em homens do que em mulheres, embora não tenha sido estabelecida predileção racial ou genética definida. O rim em ferradura frequentemente se associa a outras anomalias congênitas, sendo as mais comuns as anomalias vasculares. Este relato descreve um caso de rim em ferradura em um cadáver masculino de 73 anos, com artérias renais triplas, artéria testicular direita aberrante originada da artéria renal ipsilateral e cálices extrarrenais bilaterais. Embora seja uma ocorrência rara, o cirurgião deve estar ciente de que essa combinação de anomalias pode representar dificuldades técnicas durante cirurgias abdominais abertas ou laparoscópicas.

## INTRODUCTION

The horseshoe kidney (HSK) is a rare abnormality, but the most common type of renal fusion, first described by Jacopo Berengario da Carpi based on his autopsy findings.^1^ It is characterized by ectopia, malrotation, and vascular changes, with reported prevalence between 1 in 600 and 1 in 400. A typical HSK has two lower poles fused across the midline in front of the abdominal aorta, just below the origin of the inferior mesenteric artery (IMA). Very rarely, the upper poles may fuse resulting in a reverse HSK.^1^ Structurally, the isthmus of an HSK may be parenchymatous or fibrous in 80% and 20% cases respectively.^2^ HSK occurs twice as commonly in males than in females.^1^ No definite genetic or racial predisposition is established, but the condition has been documented in identical twins and siblings. In the majority of cases, HSK is associated with other congenital anomalies, the most common being vascular anomalies. This report describes a case of HSK with vascular variations and extra-renal calyces.

No ethical clearance was required from the local institutional ethical committee to carry out this study. The present study was carried out on a cadaver. The cadaver used in the study was donated to the department with written and informed consent for carrying out whole body dissection for education and research purposes. All norms related to the use of human cadavers in teaching and research were followed strictly as per the institutional guidelines. We also declare that the manuscript has been prepared in accordance with the Helsinki Declaration.

## CASE DESCRIPTION

**Figure 1:**
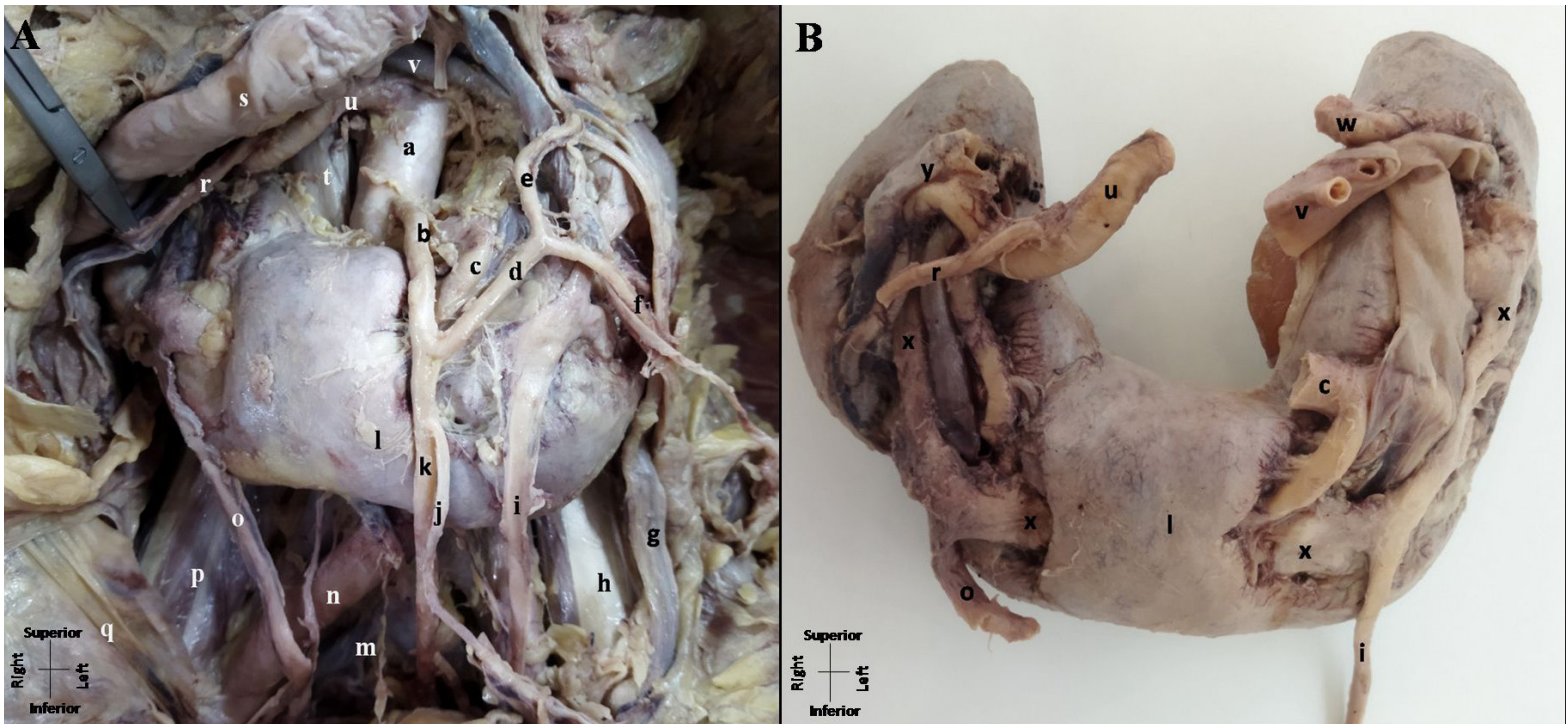
**A)** Image of the Horseshoe kidney in the cadaver and; **B)** Horseshoe kidney with ureters and renal vessels taken out of the body. a- abdominal aorta, b- inferior mesenteric artery, c- segmental branch of accessory left renal artery, d- left colic artery, e, f- ascending and descending branches of the left colic artery, g- left testicular vessels, h- left psoas major, i- left ureter, j- sigmoidal artery, k- superior rectal artery, l- isthmus of the horseshoe kidney, m- right common iliac vein, n- right common iliac artery, o- right ureter, p- right psoas major muscle, q- reflected anterior abdominal wall, r- right testicular artery arising from right renal artery, s- small intestinal loops, t- inferior vena cava, u- right renal artery, v- left renal vein, w- main left renal artery, x- extrarenal major calyces.

**Figure 2:**
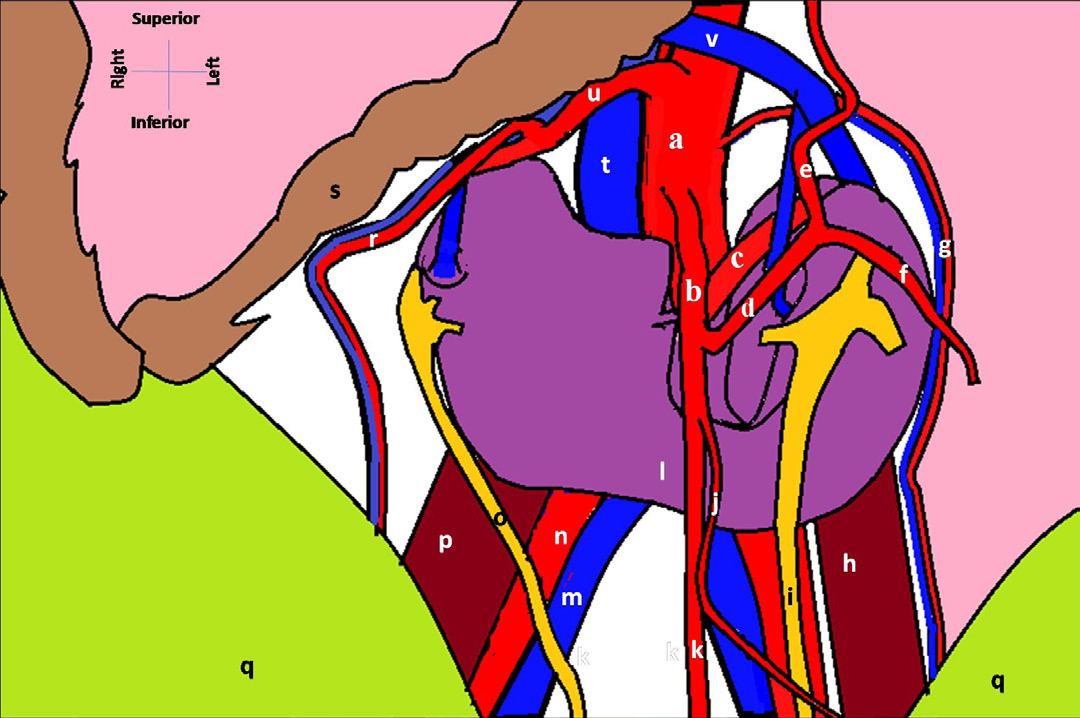
Illustration of horseshoe kidney in situ. a- abdominal aorta, b- inferior mesenteric artery, c- segmental branch of accessory left renal artery, d- left colic artery, e, f- ascending and descending branches of the left colic artery, g- left testicular vessels, h- left psoas major, i- left ureter, j- sigmoidal artery, k- superior rectal artery, l- fused lower poles of the kidneys (isthmus), m- right common iliac vein, n- right common iliac artery, o- right ureter, p- right psoas major muscle, q- reflected anterior abdominal wall, r- right testicular artery arising from right renal artery, s- small intestinal loops, t- inferior vena cava, u- right renal artery, v- left renal vein.

A horseshoe kidney with multiple vascular variations and extra-renal calyces was observed during routine dissection of the cadaver of a 73-year-old Indian male patient who died of cardiac arrest. Careful inspection and dissection of the region revealed that the HSK was retroperitoneal, lying in the lower lumbar region opposite the 2^nd^ to 4^th^ lumbar vertebrae. Three renal arteries (RAs) were seen supplying the HSK: a single right RA originating at the L2 level, and two left RAs arising at the L1 and L3 vertebral levels. Additionally, the right testicular artery was found to originate from the right RA near the right renal hilum. The IMA was seen to originate from the abdominal aorta at the L3 level, descending anterior to the fused isthmus (Figure 1 and Figure 2). Bilateral renal and testicular venous drainage was found to be normal. The renal hila of both the renal masses were seen to be directed anteriorly and the isthmus was found to be at the L3-L4 vertebral level. Two major calyces were seen forming the renal pelvis on each side. All the major and minor calyces bilaterally were found to be extra-renal. The renal pelves on either side were seen descending anterior to the renal mass, further continuing into their ureters and draining into the urinary bladder. The various dimensions of the HSK measured with Vernier Calipers are presented in Table 1.

**Table 1:** Morphometric data of the horseshoe kidney (HSK).

**Parameters**	**Right renal mass**	**Left renal mass**	**Isthmus**
Length (cm)	12.6	12.4	4.7
Width (cm)	4.6	5.1	3.8
Thickness (cm)			2.9
(Upper end)	3.5	2.9	
(Lower end)	2.6	2.7	

## DISCUSSION

### Embryology of the horseshoe kidney

The urinary system develops from the intermediate mesoderm as a nephrogenic cord (part of the urogenital ridges). The interaction between the ureteric bud (an outgrowth from the caudal part of the mesonephric duct) and the metanephric blastema during the fifth week marks the beginning of definitive kidney embryogenesis. The metanephric mesenchyme induces the ureteric bud to undergo a series of repeated branchings that eventually form the renal collecting ducts, the renal tubules, and the pelvi-calyceal system. The reciprocal induction of the metanephric blastema by the ureteric bud leads to formation of the metanephric tubules. The lumens of the metanephric tubules and the ureteric bud unite to form the nephrons. The lower poles of the developing kidneys initially lie in the fetal pelvis, with their hila facing anteriorly.^3^ The ascent of the kidneys from their pelvic position to the final adult position along the posterior abdominal wall of the lumbar region takes place approximately during the 7^th^ through 8^th^ weeks of intra-uterine development. It may so happen that the lower poles lying very close to each other fuse during the ascent and result in an HSK. The ascent of such an HSK is arrested by the inferior mesenteric artery (IMA), and the malformed kidney remains in the lower lumbar region. The midline region of the fused kidney is known as the isthmus and the ureters descend anterior to it. The ascent of the kidney is accompanied by the sequential disappearance of the caudal arteries and the successive appearance of new arterial branches to supply the definitive kidney in its final location.^1,4^ These sequential arteries may be branches of the common iliac, direct branches of the abdominal aorta, or branches of any other major artery. The ascent of the kidney is accompanied by a simultaneous 90^o^ inward rotation of the organ around its long axis, due to which the initially anteriorly-directed hila and the renal pelves eventually face medially. Hence a combination of developmental anomalies or defects in location, rotation, and vasculature are observed in HSK.^4^

The exact mechanisms responsible for renal fusion anomalies are still not very clear. Several theories have been propounded to explain the mechanism underlying the formation of HSK. The ‘*mechanical theory*’ states that the developing kidneys ascend up into the gap between the arterial forks formed by the two fetal umbilical arteries. Subsequent positional change of the umbilical arteries approximates the lower poles of kidneys which may then fuse. The ‘*abnormal caudal rotation theory*’ suggests that renal fusion is the result of changes in spatial orientation of the developing nephrogenic blastema and the ureteric bud, due to lateral flexion and rotation of the caudal end of the fetus. The ‘*ureteral theory*’ states that the HSK is formed due to induction of the contralateral metanephric blastema by a wandering ureteric bud. The ‘*teratogenic theory*’ claims the fusion anomaly to be due to the abnormal migration of posterior nephrogenic cells to the region of the isthmus due to a teratogenic insult.^5^ The *teratogenic theory* also uses this explanation to justify the increased occurrence of malignancies in HSK.^1^ The ‘*genetic theory*’ claims that disruption of the sonic hedgehog (SHH) gene signaling results in abnormal positioning of the developing renal masses which undergo subsequent fusion. HSK has also been reported in approximately 7% of individuals with Turner’s syndrome.^3^

The surplus arteries often observed in association with HSK are the persistent fetal arteries supplying the kidney in-utero. The present case exhibits one right RA and two left RAs and an aberrant right testicular artery from the right RA. All the RAs were seen to be direct branches of the abdominal aorta (Figure 1 and Figure 2). This pattern resembles type 1c of the RA classification system proposed by Natsis et al.^1^ Some of the noteworthy studies on HSK, reporting aberrant RAs and other remarkable vascular anomalies, are presented in Table 2. One important vascular anomaly associated with HSK that can have grave consequences if undetected is the abdominal aortic aneurysm (AAA). AAAs are more prevalent in the elderly and almost always fatal if ruptured. It has been estimated that ruptured AAAs are responsible for up to 55% of the mortality associated with all aortic aneurysms. The updated guidelines of the Brazilian Society for Angiology and Vascular Surgery 2023 provide specific recommendations for the management of AAA associated with HSK. The current recommendation is to undertake either open surgical repair or endovascular repair. Although endovascular repair is successful in expert hands, the guidelines recommend open surgical repair using a retroperitoneal incision as the treatment of choice in these cases. The main advantages of using a retroperitoneal incision in open surgical repair include a shorter period of adynamic ileus, early initiation of enteral feeding, and a lower incidence of incisional herniae. Moreover, retroperitoneal incisions also avoid iatrogenic damage to any accessory renal arteries and/or other aberrant visceral branches of the aorta that frequently coexist with the HSK.^21^ At times, one or multiple renal arteries may originate from the aneurysmal sac. Such an association requires prompt detection and urgent repair.

**Table 2:** Renal and other vascular anomalies reported in association with HSKs.

**Authors**	**Population and number of cases**	**Associated renal vascular / other vascular anomalies**
Chen H et al. 2001^6^	Japanese, 1 cadaveric case	In addition to normal RAs, 4 surplus arteries seen to supply the isthmus and lower part of the HSK.
Mohanty C et al. 2002^7^	Indian, 1 cadaveric case	Bilaterally, all major and few minor calyces found extra-renal; 2 small caliber RAs seen to supply upper segments on either side; additional accessory artery seen arising from right side of aorta, seen as supplying the isthmus and middle segments.
Nakamura Y et al. 2005^8^	Japanese, 2 cadaveric cases	1^st^ case: 1 right, 2 left RAs and an additional artery to the isthmus, all seen arising from the aorta 2^nd^ case: 6 RAs arising from aorta seen to supply the HSK.
Gupta M et al. 2007^9^	Indian, 1 cadaveric case	In addition to normal RAs, 2 accessory RAs from the aorta seen to supply lower segments and isthmus.
Apinhasmit W et al. 2012^10^	Thai, 1 cadaveric case	In addition to normal RAs, 6 surplus arteries seen to supply the HSK.
Naveena S and Mrudula C 2013^11^	Indian, 1 cadaveric case	In addition to normal RAs, a surplus artery seen to supply the isthmus.
Nikumbh RD et al. 2014^12^	Indian, 1 cadaveric case	Two additional arteries from the aorta seen to supply left renal mass.
Iwanaga J et al. 2016^13^	Japanese, 1 cadaveric case	Four surplus RAs observed: three branched from aorta, one seen arising from the point of bifurcation of the right common iliac artery.
Suwannakhan A and Meemon K 2016^14^	Thai, 1 cadaveric case	6 additional RAs, all arising from the abdominal aorta; renal ascent found to be arrested by an additional RA, not by inferior mesenteric artery.
Bokan RR and Shyamkishore K 2017^15^	Indian, 1 cadaveric case	Additional RA originating from the aorta seen to supply the isthmus.
Mote D et al. 2017^16^	Indian, 1 cadaveric case	In addition to normal RAs, 3 accessory arteries seen to supply right renal mass, 2 accessory arteries seen to supply left renal mass.
Lucaciu OC et al. 2019^17^	Canadian, 1 cadaveric case	An additional artery originating from the aorta seen to supply the isthmus.
Satyanarayana N et al. 2020^18^	Malaysian, 1 cadaveric case	In addition to the normal RAs, an accessory artery arising from the aorta seen to supply lower poles of kidneys and isthmus.
Coelho GM et al. 2020^19^	Brazilian, 1 clinical case	A horseshoe kidney with a huge abdominal aortic aneurysm was detected in 65-year-old male smoker wherein the HSK was supplied by four renal arteries, two of which were arising from the aneurysmal aortic sac.
Lee YE et al. 2023^20^	American, 1 cadaveric case	Co-existence of an HSK with abdominal aortic aneurysm observed in a male cadaver.

It is very rare for both the testicular arteries to arise from arteries other than the abdominal aorta, however unilateral aberrant origin of the testicular artery is known. Several types of origin of testicular arteries from the RA are reported in literature. Kotian et al.^22^ classified testicular arteries based on their origin. The origin of testicular arteries from renal arteries is classified as Type III. However, a sub-classification of testicular arteries of renal artery origin is not available. When the testicular artery arises from an RA, the origin may be from the main RA, a segmental RA, or an accessory RA. Some important studies documenting the origin of testicular arteries from RAs are presented in Table 3.

**Table 3:** Studies documenting the origin of testicular arteries from renal artery.

**Author**	**Sample size**	**Population**	**Right sided renal artery**	**Left sided renal artery**	**Total incidence**
Terayama H et al. 2021^23^	27	Japanese	-	2	2 (7.4%)
Kotian SR et al. 2016^22^	42	Indian	-	4	4 (9.52%)
Hussein M et al. 2014^24^	33	Indian	11	5	16 (48.48%)
Misiani MK et al. 2012^25^	1	African	-	1	1
Mamatha H et al. 2015^26^	40	Indian	1	4	5 (12.5%)
Panagouli E et al. 2012^27^	77	Greek	1	1	2 (2.59%)

The present case of HSK also exhibited extra-renal calyces (ERC), a very rare condition wherein major and minor calyces of the kidney lie outside the renal hilum. HSK has been reported in association with an extra-renal calyceal system. The occurrence of ERC has been considered to be due to the disparity resulting from slow development of the ureteric bud, permitting extra-renal development of the first or second order channels of the collecting system.^1^ In these cases of HSK with ERC, the uretero-pelvic junction is pulled upwards, predisposing to uretero-pelvic obstruction and subsequent urinary stasis, calculus formation, and infection. We found bilateral ERC in the present case – an extremely rare anomaly – with two major calyces joining outside each renal mass to form a very small renal pelvis which continued as ureters on either side.

HSK is usually an incidental finding while performing imaging studies of the abdomen and kidney-ureter-bladder region. It may be detected in a plain radiograph of the abdomen, because the perinephric fat surrounding the HSK casts a soft tissue shadow. Furthermore, the renal masses lie at the lower lumbar region and their long axes are seen closer to the spine. For a definitive diagnosis, a computed tomography (CT) scan and scintigraphy should be performed. Digital subtraction angiography (DSA) may provide valuable information to identify aberrant RAs and to understand the relation of the HSK with its vasculature. The vascular anatomy of the HSK can be better determined with CT and magnetic resonance imaging (MRI). 3D volume-rendered CT angiography locates and delineates vessels more precisely than conventional angiography.

HSK may remain unnoticed throughout life, as in the present case. The ERCs may also not cause any clinical problems.^1^ However, the presence of additional RAs and ERC in conjunction with HSK definitely has clinical relevance. Several clinically relevant genitourinary anomalies are associated with HSK. Vesico-ureteric reflux can be associated with HSK and can be treated conservatively in 8-32% cases but may require surgical intervention in 25% of cases. If HSK is associated with pelvic-ureteric junction obstruction, pyeloplasty is usually required in frank symptomatic cases. About 20-80% of HSKs are found to be associated with renal stone diseases. Stones smaller than 15 mm and not situated in the lower pole can be approached with short wave lithotripsy (SWL) or ureteroscopy (URS). Stones that fail treatment with SWL or URS and stones larger than 15 mm should be considered for percutaneous nephrolithotomy (PCNL). Pre-procedural imaging, such as CT scanning, is essential during the workup to prevent accidental bowel injury in these types of cases.^28^ If HSK is associated with any type of renal cancer, management is the same as for normal patients with renal cancers. Nonetheless, the incidence of Wilms’ tumor in horseshoe kidneys is 1.76 to 7.93 times higher than that expected in the general population. The use of computerized 3D image modeling can help dramatically improve surgical decision-making and preoperative treatment planning as well as assist in tailoring of the surgical approach and strategy in complex anatomical cases such as HSK that require surgical intervention.^29,30^ Kidney transplant from cadaveric donors is a reality today. There are also several known cases of successful cadaveric HSK transplantation. Owing to the frequent occurrence of aberrant vasculature associated with HSK, harvesting a horseshoe kidney may prove difficult in the absence of sufficient knowledge about possible vascular variations associated with this developmental anomaly.

## CONCLUSION

This report describes a horseshoe kidney with triple renal arteries and aberrant right testicular artery originating from the ipsilateral renal artery and bilateral extra-renal calyces. Although rare in occurrence, surgeons should be aware of such a constellation of anomalies associated with horseshoe kidneys, since their identification presents a unique challenge and surgical outcomes may be determined by their detection and management.
